# Sensing and Responding to UV-A in Cyanobacteria

**DOI:** 10.3390/ijms131216303

**Published:** 2012-12-03

**Authors:** Yoon-Jung Moon, Seung Il Kim, Young-Ho Chung

**Affiliations:** Division of Life Science, Korea Basic Science Institute, Daejeon 305-806, Korea; E-Mails: moonyj@kbsi.re.kr (Y.-J.M.); ksi@kbsi.re.kr (S.I.K.)

**Keywords:** ultraviolet-A, cyanobacteria, photoreceptor, signaling pathway, negative phototaxis, pterin, reactive oxygen species, chromophore, programmed cell death, caspase

## Abstract

Ultraviolet (UV) radiation can cause stresses or act as a photoregulatory signal depending on its wavelengths and fluence rates. Although the most harmful effects of UV on living cells are generally attributed to UV-B radiation, UV-A radiation can also affect many aspects of cellular processes. In cyanobacteria, most studies have concentrated on the damaging effect of UV and defense mechanisms to withstand UV stress. However, little is known about the activation mechanism of signaling components or their pathways which are implicated in the process following UV irradiation. Motile cyanobacteria use a very precise negative phototaxis signaling system to move away from high levels of solar radiation, which is an effective escape mechanism to avoid the detrimental effects of UV radiation. Recently, two different UV-A-induced signaling systems for regulating cyanobacterial phototaxis were characterized at the photophysiological and molecular levels. Here, we review the current understanding of the UV-A mediated signaling pathways in the context of the UV-A perception mechanism, early signaling components, and negative phototactic responses. In addition, increasing evidences supporting a role of pterins in response to UV radiation are discussed. We outline the effect of UV-induced cell damage, associated signaling molecules, and programmed cell death under UV-mediated oxidative stress.

## 1. Introduction

Ultraviolet light A (UV-A) (315–400 nm) comprises the largest portion of the total solar UV reaching the earth [1], since all the UV-C (~180–280 nm) and most of the extraterrestrial ultraviolet B (UV-B) (280–315 nm) are absorbed by the earth’s stratospheric ozone layer. Although UV-B radiation accounts for less than 1% of the total energy reaching the Earth’s surface, it is a highly active component of the solar spectrum and can have significant effects on the biota [2,3]. Increased exposure to UV-B irradiance caused by the depletion of the ozone layer is potentially detrimental to many photosynthetic microorganisms in aquatic and terrestrial ecosystems [4,5]. Currently, UV-A, like UV-B, is considered to be a crucial environmental signal for which photoautotrophic microorganisms have evolved specific, sensitive UV perception systems and avoidance strategies [6,7]. Thus, both UV-A and UV-B are known to directly or indirectly affect the biological activities of algae or cyanobacteria [8,9]. UV-B has the greatest potential for cell damage, which is caused by both direct effects on DNA and proteins, as well as indirect effects via the production of reactive oxygen species [10,11]. UV-B radiation has been shown to have a negative effect on various physiological and biochemical processes, such as photosynthesis [12,13], growth [14], pigmentation [15,16], nitrogen metabolism [17,18], CO_2_ uptake and ribulose 1,5-bisphosphate carboxylase/oxygenase (RuBisCO) activity [19,20]. It can also impair the motility and photoorientation of cells, and alter the morphology of filamentous cyanobacteria [21,22]. UV-A irradiation mainly has indirect effects via energy transfer from UV-A stimulated chromophores to the DNA target, or via the photosensitized production of ROS [1,11]. In addition, UV-A induces direct damage to photosystem II via the same mechanism as UV-B does [23]. Under normal ozone conditions, UV-A is the main inhibitory factor of photosynthesis in phytoplankton [24]. Cyanobacteria, like plants and algae, have developed physiological mechanisms allowing acclimation and survival in harmful UV irradiation conditions. Their protection strategies include stress avoidance by migration, stress defense by induction of UV-absorbing molecules as well as antioxidants, and repair mechanisms including DNA repair and *de novo* synthesis of D1 and D2 proteins for the repair of the damaged photosystem II complex [25–30]. While much research has focused on the effects of UV-B radiation, little is known about UV-A-mediated signaling processes and their roles in the stress responses of photosynthetic microorganisms. UV-A radiation affects many phenomena negatively, but the exact mechanism is still elusive. Therefore, the studies of UV-A-induced signal transduction will help in understanding the defense mechanisms employed by the cyanobacteria which enable them to cope with harmful UV radiation. Several comprehensive reviews are presented, in detail, on cyanobacteria and protection mechanisms against the detrimental effects of UV [6,31,32]. In this review, we focus predominantly on recent progress in elucidating the UV-A signaling pathway, including UV-A perception mechanism and phototactic responses in cyanobacteria. This review also provides a possible function of pterins in cyanobacteria, namely as a chromophore of UV-A photoreceptor systems. Finally, we discuss the effects of UV irradiation on cyanobacteria, as well as their programmed cell death to counteract UV damage in view of UV-sensing and signaling under UV-mediated oxidative stress.

## 2. UV-Mediated Signaling Pathways in Cyanobacteria

**Figure 1:**
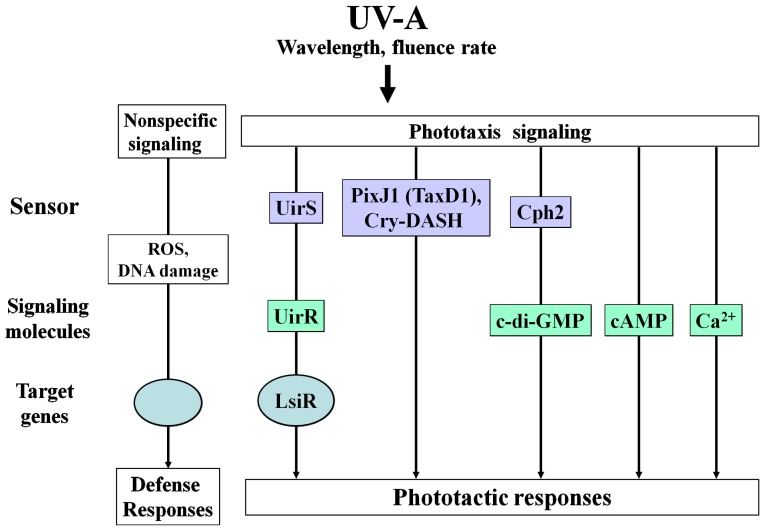
Schematic depiction of the different classes of UV-A sensors, signaling molecules and the UV-A signaling pathways in which they are involved. UV-A stimulates distinct nonspecific and UV-A specific phototaxis signaling pathways, depending on the wavelengths and fluence rates, leading to the induction of specific target genes and downstream responses in cyanobacteria.

**Figure 2:**
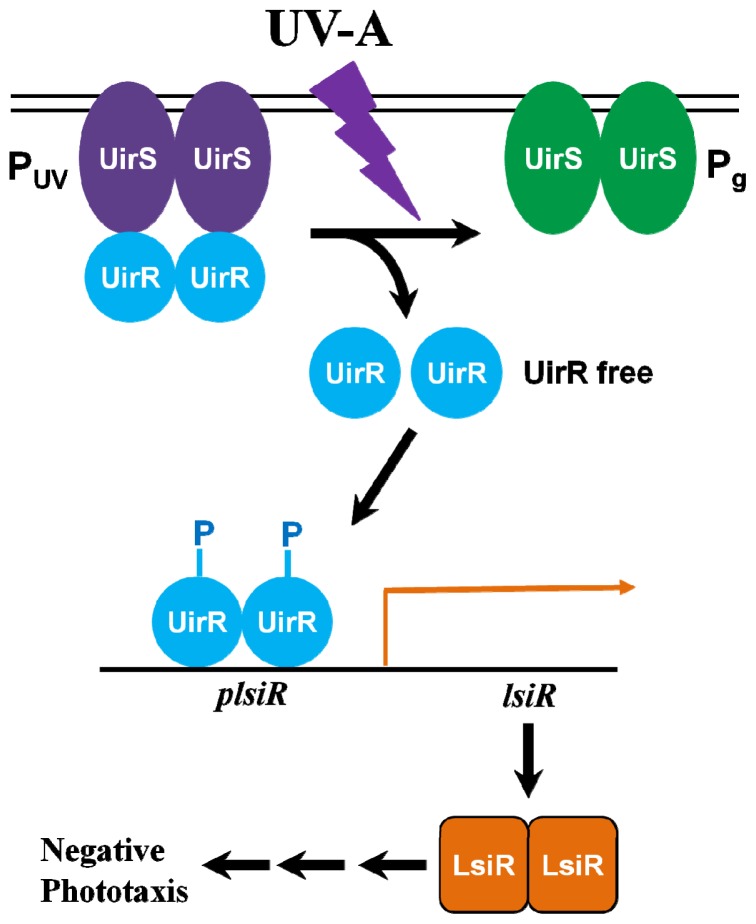
Schematic illustration of negative phototaxis signaling pathways induced by high fluence rate UV-A irradiation. Figure modified from Reference [60].

**Figure 3:**
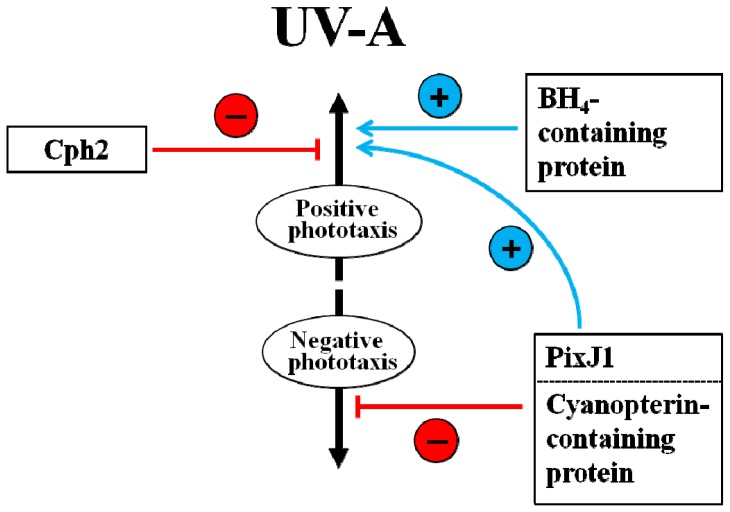
A possible model showing the regulatory roles of Cph2, PixJ1 and pterin-related photoreceptor protein(s) in the low fluence rate UV-A-induced phototaxis signaling pathway of cyanobacterium *Synechocystis* sp. PCC 6803. Figure concept adapted from Reference [57].

Light is not only a source of energy for photosynthetic lifeforms, but also causes cellular damage through a range of mechanisms. Phototactic responses enable photosynthetic microorganism to migrate towards environmental niches in which light is bright enough to efficiently drive photosynthesis, while at the same time being sufficiently filtered of harmful UV or excessive light [33,34]. Photosynthetic microorganisms containing endogenous photosensitizer(s) use defensive pigment(s) against harmful light, as relatively high intensity light can cause severe damage, and hence, their capability of avoiding such exposure is directly linked to their survival. To escape from noxious light environments, photosynthetic microorganisms adopt at least three behavioral strategies based on directional (phototaxis) as well as non-directional (photophobic responses, photokinesis) movements [35,36]. Phototaxis is dependent on light direction, and can be either positive (movement toward the light source) or negative (movement away from the light source) [37]. Cyanobacteria are oxygen producing photosynthetic prokaryotes, and possess many photosensing systems for adaptation to changes in their light environment. In particular, the unicellular cyanobacterium *Synechocystis* sp. PCC 6803 exhibits type IV pilus-dependent phototaxis in response to unidirectional light [38–40]. Cyanobacteria respond to a broad spectrum of light, ranging from near-UV to far-red light [41,42]. In order to accurately perceive the light environment and prevent damage caused by toxic light exposure, cyanobacteria possess several classes of photoreceptors, such as phytochromes, UV-A/blue photosensors, and as yet undefined photoreception systems of mediating responses to UV-B [41,42]. Cyanobacteria can sense light intensity, different spectral ranges, and the direction of light using these highly specialized photoreceptors. Of these, cyanobacteriochromes (CBCRs) are well-characterized phytochrome-related photoreceptors. Phytochrome-related CBCRs exhibit unique and diverse photochemical properties, which are distinct from the red/far-red reversible photoconversion of plant phytochromes [43]. Photoactive GAF domains of CBCRs were found to show reversible photoconversion between blue and green [44–46], green and red [47–50], UV and blue [51] or unidirectional photoconversion from violet to yellow absorbing states [52]. Considerable numbers of phytochrome-related CBCRs have been reported to be implicated in light-dependent physiological responses such as chromatic adaptation [48,49,53] and phototaxis [54]. Nevertheless, physiological roles of many other candidates for phytochrome-related CBCRs remain to be elucidated. UV signaling is an important but poorly understood aspect of light responsiveness in cyanobacteria on the molecular biological level. Although several genes encoding photoreceptors, and the primary molecular events have been identified, the natures of molecules acting as UV sensors and the downstream signaling mechanisms have, thus far, not been elucidated. The model cyanobacterium *Synechocystis* sp. PCC 6803 (hereafter Syn6803), utilizes more than three distinct classes of photosensors to control UV-A-induced phototaxis, the phytochrome-related cyanobacteriochromes (CBCRs) including PixJ1 (or TaxD1) [54,55] and Cph2 [56,57], Cry-DASH Sll1629 [58,59], and the ETR1-related UV intensity sensor (UirS) Slr1212 [60] (Figure 1). Genetic screens and photophysiological approaches of pterin biosynthesis gene [58,59] and cyanobacteriochromes [57,60] now present an opportunity to advance our understanding of how cyanobacteria process the UV-A signal. UV-B is a highly variable environmental signal, and fluctuations in fluence rates will probably modulate the levels of CPDs (cyclobutane pyrimidine dimers), ROS (reactive oxygen species), and photoprotective signaling molecules, as well as the induction of UV-stress proteins [11]. UV-A also has similar effects to those of UV-B, but with less consequence [7,61]. In cyanobacteria, UV-A also induces oxidative stress and lipid peroxidation, and damages photosynthetic apparatus, decreasing survival and inhibiting growth [7,61]. Recent studies have shown that separate UV-A signaling pathways may function optimally at different UV-A fluence rates [57,58,60]. UV-A-induced phototactic responses in cyanobacteria are classified according to the fluence rates required to initiate them (Figures 2 and 3). The two UV-A signaling pathways, as well as the genes they regulate, are represented diagrammatically in Figures 2 and 3.

### 2.1. High Fluence Rate UV-A Dependent Phototaxis Signaling Pathway

Recently, it has been shown that wild type Syn6803 cells exhibited non-motility on agar plates under low fluence rates, and negative phototaxis as UV-A fluence rates were increased [57,58,60]. When cyanobacterial cells face high fluxes of UV radiation, negative phototactic response functions are a first line of defense for the avoidance of UV-induced damage and oxidative stress. Since cyanobacteria contain photosynthetic pigments (e.g., chlorophylls and phycobilins), which act as potential photosensitizers under UV, they are prone to oxidative stress [11,62]. Exposure of cyanobacterial cells to UV-A radiation has been found to cause oxidative stress by producing ROS via photosensitized reactions [62,63]. In plants, high fluence rates of UV-B are known to generate ROS and to increase levels of signaling molecules which are involved in defense responses [64,65]. It has been suggested that the increased production of ROS and the resultant oxidative stress under UV are considered to be the initial event, and may act as an alert signal to regulate genes that induce protective response at the transcriptional and translational levels [11,64,65]. Cyanobacteria seem to show diverse responses to UV, ranging from acute stress responses to regulatory phototactic responses. Our understanding of the mechanisms of cyanobacterial phototactic response to UV-A radiation has been greatly advanced by the identification of UirS (UV intensity response sensor, Slr1212) and UirR (UV intensity response regulator, Slr1213)-LsiR (light and stress integrating response regulator, Slr1214) as primary UV-A photoreceptor responses to high fluence rates of UV-A [60]. Through a combination of biochemical and molecular genetic analysis, the possible function of UirS, and its cognate response regulator UirR, in UV-A defense mechanisms was characterized [60]. From the analysis of protein interactions and photophysiological responses, Song *et al*. proposed that UirS acts as a UV-A photosensor and induces the expression of LsiR via release of bound UirR, which targets the LsiR promoter. Similar to other light sensors belonging to the CBCRs family [44–46], UirS possess two conserved cysteine residues within its phycocyanobilin (PCB)-binding GAF domain [60,66]. *N*-terminal regions containing transmembrane (TM) helices of UirS showed close similarity to the ethylene-binding domain of the *Arabidopsis* ethylene receptor (*ETR1*), and was suggested to be responsible for ethylene binding [67]. On the other hand, its PCB-binding GAF domain was found to be required for generation of the blue/green light photoreversible CBCR [68]. Based on its structural organization, it has been proposed that UirS may function as a hybrid receptor to simultaneously integrate light and hormone signals [68]. UirR and LsiR belong to the AraC family of transcription activators of stress response, and PatA-type response regulators, respectively. In previous reports, PatA-type response regulators in Syn6803 were shown to be induced by diverse stresses, such as inorganic carbon and iron limitations [69,70], exposure to H_2_O_2_[71], and high light intensities [72]. At high fluence rates of UV-A, a light signal is likely to be perceived by the membrane-bound UirS-UirR two-component signaling system, and is then likely to be transduced through phosphorylation of UirR, which activates LsiR transcription to regulate the negative UV-A phototaxis signaling pathway (Figure 2). Thus, LsiR functions as a signal output regulator of negative phototaxis under high UV-A fluence rates. Based on two-hybrid experiments, direct interactions and possible phospho-transfers from the UirS-UirR two-component system to LsiR were proposed. It is possible that phosphorylation is required for UirR to function as an activator of *lsiR* transcription, and thus, these three proteins together with another histidine kinase (HK) could form a phosphotransfer relay cascade [60]. At high fluence rates of UV-A, UirR activation by phosphorylation might depend on ROS generation, particularly the increased intracellular hydrogen peroxide (H_2_O_2_) which has been associated with the phosphorylation of the protein kinase domain in plants [73,74], although the precise mechanism of UV-A irradiation induced-UirR activation has not yet been elucidated. Alternatively, the UirS-UirR two-component signaling system may have a dual function in Syn6803, that of perceiving high intensity UV-A, as well as acting as a target for ROS (e.g., H_2_O_2_), thereby mediating the downstream signaling process to initiate UV-induced negative phototaxis, in a similar manner to the plant ethylene receptor *ETR1*[75,76]. In *Arabidopsis*, oxidative modifications of reactive Cys residues within the *N*-terminal domain of *ETR1* has been suggested as a means by which H_2_O_2_ signaling can activate cellular responses such as gene expression and reversible protein phosphorylation [74,77]. Although the histidine kinase function of *ETR1* was not shown to be required for H_2_O_2_-induced stomatal closure, the *N*-terminus of *ETR1* appears to be essential for H_2_O_2_ mediated signaling process [75]. Besides *ETR1*, the two-component signaling system of plants appears to be involved in several other signaling pathways [78]. For instance, recent research has revealed a functional cross-talk between cytokinin (a hybrid histidine kinase) and light (phytochrome B) signaling [79,80]. Presumably, the UirS-UirR-LsiR two-component-based UV-A phototaxis signaling pathway may be closely linked to ROS signaling, as seen in ethylene and H_2_O_2_ cross-talk induced stomatal closure in *Arabidopsis*[75,76]. This can be further supported by the fact that high fluence rate of UV-B response in plants share gene activation and signal transduction pathways which are commonly associated with other biotic and abiotic stresses [65,81,82]. Similar to the findings made for plant, functional overlap is also likely to exist between ROS and the UirS-UirR two-component-based UV-A signaling pathway in cyanobacteria, although whether such shared responses occur in Syn6803 cells remains to be determined. At high fluence rates of UV-A, the cyanobacterial UV-A-induced signaling pathway may be interlinked with stress mediated signaling pathways. Thus, cyanobacteria may defend themselves against high fluence rates of UV-A through mechanisms which overlap with general stress-response signaling systems.

### 2.2. Low Fluence Rate UV-A Dependent Phototaxis Signaling Pathway

It was reported that low-fluence-rates of UV-B can serve as a signal to regulate plant growth and development [65]. In *Arabidopsis*, low fluence rates of UV-B are known to regulate developmental processes, including inhibition of hypocotyl and enhancement of cotyledon opening, and induction of specific sets of genes required for UV-B tolerance [65,83]. Similar to the effects of low fluence rates of UV-B on plants, low fluence rates of UV-A responses in cyanobacteria appear to be photoregulatory rather than resulting from photodamage or stress. Although low fluence rates of UV-A responses are not well characterized, there is compelling evidence, principally from studies using mutants defective in pterin biosynthesis and phytochrome-related genes, that they are distinct from stress signaling pathways. Both phytochrome-like photoreceptor Cph2 and cyanobacteriochrome PixJ1 (TaxD1) are considered to be the best known UV-A-induced signaling components. Cph2, previously known to inhibit phototaxis toward blue light in Syn6803 [56], has recently been shown to be required for the inhibition of positive phototaxis in response to low intensity UV-A [57]. Whereas wild-type showed no phototaxis under low fluence rates of UV-A irradiation, the *cph2* mutant moved towards the UV-A [57]. Because the *pixJ1* (*taxD1*) mutant showed negative phototaxis away from low fluence rates of UV-A and blue light, as well as in the green to red *in vivo*[57,84], the PixJ1 (TaxD1) receptor can also function as the photoreceptor that governs positive phototaxis even in the UV-A/blue signaling pathway of Syn6803. An insight into the involvement of Cph2 and PixJ1 in the regulation of UV-A-induced phototaxis was obtained through the demonstration that *pixJ1* mutation is epistatic to *cph2* under UV-A. It was shown that the phenotype of the *cph2*/*pixJ1* double mutant resembled that of the *pixJ1* mutant, indicating that PixJ1 and Cph2 may act through the same pathway as PixJ1, being downstream of Cph2 [57]. Thus, additional interaction between Cph2 and PixJ1 (Tax D1) might be important for light-induced phototaxis, as documented in previous reports [56,84]. The induction of positive phototaxis was also proposed to depend on the combined effect of regulatory activities of these two phytochrome-like proteins and the UV-A/blue photosensor [57,58]. In accordance with this view, the involvement of positive phototaxis of tetrahydrobiopterin (BH_4_)-containing protein in UV-A may be one aspect of the concerted action with PixJ1 photoreceptor. Using studies with pterin biosynthesis inhibitor and cryptochrome knockout mutant, it was shown that in contrast to the negative regulation of Cph2 in positive phototaxis, BH_4_-containing protein probably acts as a positive regulator and in concert with phytochrome-like PixJ1 (TaxD1) photoreceptor, to induce positive phototaxis in the UV-A signaling pathway of Syn6803 [58]. Since phytochrome-like photoreceptor PixJ1 (or TaxD1), BLUF photosensor PixD is involved in positive phototaxis, the existence of an additional photoreceptor involved in modulating negative phototaxis has also been proposed [54,84–86]. It has been recently reported that Cry-DASH Sll1629 may participate in the perception of UV-A/blue light to inhibit negative phototaxis in Syn6803 [58,59]. Under a relatively low fluence rate of UV, the inhibitory effect of Cph2 on positive phototaxis could be counterbalanced by the opposite action of cyanopterin-containing protein (Figure 3). Under UV-A, the opposite effects of Cph2 and cyanopterin-containing protein on phototaxis suggest that Syn6803 may employ an elaborate controlling mechanism to sense UV-A, using different photoreceptor component or regulators. However, despite these recent advances in our understanding of the different signaling pathways, many aspects related to UV-A perception still remain unclear. Although low fluence rate UV-A responses appear to be photoregulatory rather than resulting from damage or stress, there is little information as to how UV-A activates components of the signaling pathways.

### 2.3. Second Messengers in Cellular Response to UV Radiation

c-di-GMP appears to be involved in the regulation of cyanobacterial phototaxis. Recently, it was shown that expression of the CBCR-GGDEF2 module in Δ*cph2* mutant cells produced c-di-GMP under blue light irradiation, leading to the inhibition of phototaxis [87]. In addition, high levels of c-di-GMP were shown to inhibit flagellar and pili-based motility in several species of bacteria [88–90]. Phytochrome-related CBCRs can sense a very broad spectral range extending from the near UV to the far red region [43,66] and are considered to translate this information into changes of c-di-GMP levels [87]. Accordingly, increased c-di-GMP levels are presumed to be responsible for inhibiting phototaxis toward low intensity UV-A (Figure 1). Further specific cellular targets of c-di-GMP need to be identified.

Under UV stress conditions, changes in intracellular cGMP levels were found to be involved in the signal transduction process which permits the repair of UV-B-damaged PSII in Syn6803 [91]. Inactivation of *slr2100*, encoding putative cGMP phosphodiesterase, resulted in a higher level of intracellular cGMP, which led to accelerated photoinhibition under UV stress.

cAMP may also act as a second messenger in the signal transduction for phototactic responses to UV-A. The cellular cAMP level was found to increase in response to UV-A (380 nm) as well as blue (450 nm) light, which led to the stimulation of cell motility [92]. In Syn6803, both cAMP and its receptor protein (SYCRP1) are involved in biogenesis of the pili required for cell motility [93,94]. Inactivation mutants of either the adenylate cyclase (*cya1*) or cAMP receptor gene (*sycrp1*) are indeed non-motile, and externally added cAMP restored only the motility of *cya1* mutant [95,96]. Thus, it was noted that in the absence of the receptor protein, the exogenous addition of cAMP had no effect on motility [96]. In addition, the blue light induced photoavoidance response in *Euglena gracilis* was found to be mediated by a photoactivated adenylyl cyclase, PAC [97]. These results indicate that cAMP produced by Cya1 might mediate a UV-A/blue light signal to stimulate cell motility. A previous study reported that BLUF photosensor (Slr1694) and cyanobacterial cryptochrome Cry-DASH (Sll1629) do not appear to be involved in the regulation of Cya1-mediated cAMP signal transduction in response to blue light [92,98]. Thus far, it is not clear whether cryptochrome-like proteins control phototaxis through cAMP-mediated signal transduction in response to UV-A.

In plants, ROS is an important component of the defense response to various environmental stresses [65,73]. ROS are produced not only under UV, but also induced by visible light which is the main component of natural sunlight, and produced in several places under normal functioning of the photosynthetic apparatus (PSI and PSII) and in the respiratory electron transport chain. Excessive production of ROS causes oxidative damage or the apoptotic cell death. During evolution, plants have evolved to develop an antioxidant defense system against oxidative stress damage [99]. ROS and, more particularly, H_2_O_2_, play key roles in a broad range of physiological processes [100]. Recently, H_2_O_2_ has also been regarded as an important signal governing the expression of various genes, including antioxidants, photosynthetic genes, and signaling proteins such as kinase, phosphatase, and transcription factors [64,65,73,74]. Similarly, ROS may also act as a signal and/or second messenger enabling cyanobacteria to regulate the expression of a number of genes, resulting in protection from various environmental stresses, especially high and UV irradiance [11,101,102]. In the cyanobacterium *Anabaena* sp., UV-B-and UV-A-induced ROS production was detected *in vivo* using a ROS-sensitive probe [62,103,104]. Transcriptomic and proteomic analysis showed that UVB increases the expression of ROS-scavenging enzymes such as glutathione peroxidase and superoxide dismutase in cyanobacteria [105,106]. Exogenous addition of antioxidants such as ascorbate and *N*-acetyl cysteine (NAC) was found to reduce UV-induced oxidative damage, and led to a higher survival rate in *Anabaena* sp. [103]. Singlet oxygen generated in high light intensity has been suggested to trigger the sign reversal from positive to negative for phototaxis in *Anabaena variabilis*[107]. In the cyanobacterium *Anacystis nidulans* R-2, both near-UV irradiation and exposure to methyl viologen induced UV-shock proteins, indicating the role of ROS in transducing UV-A signals [108]. As a result, UV-induced and general oxidative stress responses are significantly overlapping at higher UV intensities as shown by the upregulation of antioxidant defense systems and heat shock proteins in cyanobacteria [105]. However, in higher plants, low fluence UV-B induces specific signaling pathways via UVR8 and COP1, which are apparently unrelated to ROS signaling [109].

Ca^2+^ signals have been regarded as a core regulator of cellular responses to various environments [110]. In the *Arabidopsis* cell culture, experiments with calcium antagonists indicate that calcium is involved in both the UV-B and UV-A/blue signal transduction pathways, regulating chalcone synthase (*CHS*) gene expression [111]. There are several findings that support the role of Ca^2+^ as an intracellular second messenger in cyanobacteria [112–114]. Intracellular free Ca^2+^ concentrations increase several fold in heterocysts and are regulated by CcbP, a Ca^2+^-binding protein found in *Anabaena*[115]. Calcium ion concentrations have been primarily considered to be important for the regulating of gliding motility and phototactic responses in cyanobacteria [116]. In addition, calmodulin-like proteins have also been characterized in cyanobacterium *Nostoc* sp. PCC 6720 [117]. Recently, comparative analysis of available *A. platensis* genomes has revealed the large number of genes encoding extracellular proteins with Ca^2+^-binding domains [118]. Also, calcium may contribute to UV signaling in cyanobacteria. Exposure of the cells to UV was found to increase cytosolic Ca^2+^ concentrations in a cyanobacterium *Anabaena* sp. [119]. UV sensitive l-type calcium channels were detected in *Anabaena* sp. and *N. commune*[120].

Thus, it is likely that cyanobacteria transduce UV signals via cAMP and calcium to regulate photoavoidance response. As described above, changes in cellular homeostasis of c-di-GMP also seem to be involved in the signal transduction for UV-induced phototactic response. However, we do not know how these different signals are integrated into the regulation of UV-dependent phototaxis signaling pathways.

## 3. Role of Pterin in Responses to UV Radiation

Pterin has been postulated as the putative candidate for the chromophore operating in association with flavin chromophore in the near UV/blue region [121]. Previously published reports have provided some basic information as to pteridine biosynthesis and photophysiological properties, which could help to unravel the photobiological and other roles of pteridines in Syn6803 [122–126]. Despite circumstantial evidences about the critical role of pterin in cyanobacteria, the question as to whether pterin could play a role in UV-A sensing within cyanobacteria remains an open question, as no systematic approach to investigate its physiological function *in vivo* has yet been determined. Therefore, our work on pterin was initiated to understand whether or not this pigment might be a part of the near UV/blue light sensing system in Syn6803. Here, we review the potential role of pterin as a chromophore of the cryptochrome considered as a possible UV-A/blue light photoreceptor in phototaxis of Syn6803.

### 3.1. Cyanobacterial Pteridines

The main part of pterins is comprised of a bicyclic ring system, the pyrazino [2,3-d] pyrimidine or pteridine moiety [121]. Most pterins occurring in nature have the 2-amino-4-hydroxypteridine structure, and various pterin derivatives occur in tetrahydro, dihydro, or fully oxidized forms [121,127]. Folic acid is an important cofactor in cellular metabolism, and it, along with its derivatives are called “conjugated” pteridines (referring to the linkage of *p*-aminobenzoylglutamate), whereas other pterins (including biopterin, molybdopterin, and pterin pigments) are referred to as unconjugated pteridines [127]. GTP is a common precursor of both tetrahydrofolate and tetrahydrobiopterin in cells which have the capacity for their synthesis. Unconjugated pteridine compounds are ubiquitous in nature as cofactors and pigments [127]. Tetrahydrobiopterin (BH_4_) is best known as a cofactor for aromatic amino acid hydroxylation and nitric oxide synthesis in higher animals [128]. Molybdopterin, which is ubiquitous within bacterial and animal life, is essential for the formations of aldehyde oxidase, nitrate reductase, sulfite oxidase, and others [127]. Besides, sepiapterin is well known as the precursor of BH_4_ synthesis [122,129].

Pteridine glycosides, which have various kinds of sugars attached to the side chain of the pterin ring at C-6, such as biopterin, 6-hydroxymethylpterin, and neopterin. Tetrahydrobiopterin (BH_4_)-glucoside, as well as several other pteridine glycosides, is produced at high cellular concentrations in cyanobacteria [130–134], although some were also found in a few prokaryotes such as the anaerobic photosynthetic bacteria *Chlorobium tepidum*[135] and *Chlorobium limicola*[136], and the chemoautotrophic archaebacterium, *Sulfolobus solfataricus*[137]. The biological functions of pteridine glycosides remain unknown, though earlier studies have suggested their possible role in photosynthetic electron transport [138]. Additionally, protection against UV damage was also proposed as a possible function, due to the finding of increased biopterin-glucoside synthesis in marine cyanobacterium, *Oscillatoria* sp., upon exposure to UV-A irradiation [139,140]. On the other hand, biopterin-glucoside was reported to be implicated in the stabilization of phycocyanin upon exposure to UV [141,142]. Pteridine glycosides are abundant and ubiquitous in cyanobacteria, implying that it has a number of essential roles.

Recently, a novel form of pteridine glycoside, cyanopterin, was identified in Syn6803 [132]. Cyanopterin was determined to have the chemical structure of 6-hydroxymethylpterin-4-*O*-methylglucuronylgalactoside [132]. Based on the chemical structure and the genome sequence, genes encoding biosynthetic enzymes for the pteridine moiety were identified [124]. The enzymes, named pteridine glucosyltransferases (hereafter PgtA) were thought to be important, not only for establishing the biosynthesis, but also for understanding the functional role of cyanopterin or its sugar moiety [126]. PgtA enzymes were considered to be important as useful targets for gene disruption studies because it was presumed that the putative function of pteridine glycosides would be conferred by the sugar attached to the pterin moiety. PgtA enzymes were initially identified from the cyanobacterium *Synechococcus* sp. PCC 7942, in which a BH_4_-glucoside was identified [125,133]. The enzyme, named UDP-glucose: BH_4_ α-glucosyltransferase (BGluT), catalyzes the synthesis of the BH_4_-glucoside by transferring glucose from UDP-glucose to BH_4_. The BGluT protein shared a high-sequence homology with the putative protein encoded by *slr1166* from Syn6803 [126]. Cyanopterin contains 6-hydroxymethylpterin as the pterin moiety [132], whereas most other pteridine glycosides have the structure of biopterin glycosides [125,140,143]. Thus, PgtA, which catalyzes the transfer of the final sugar to 6-hydroxymethylpterin in cyanopterin synthesis, is encoded by *slr1166* in Syn6803 [126]. Cyanopterin exists in the tetrahydro form *in vivo*, and is produced constitutively in large amounts, comparable to chlorophyll a, suggesting that it has some essential function in Syn6803 [132]. In addition, a mutation that disrupted the *slr1166* gene encoding *p*teridine *g*lycosyl*t*ransferase (*pgtA*) resulted in a lower intracellular pterin content and growth rate relative to the wild-type [126].

### 3.2. Evidences for the Involvement of Pterin in UV-Perception

According to the action spectra of UV-A/blue responses and their physico-chemical properties, pterins are presumed to be the chromophore of the UV-A/blue receptors [121]. The first line of evidence for the possible role of pterin in photoperception came from the studies of mutants of *Phycomyces blakesleeanus* and from two-enzyme systems that serve as photoreceptor models, *i.e.*, nitrate reductase (containing a molybdo-pterin subunit) and the microbial DNA photolyase [144–146]. In *Phycomyces* the action spectra for blue-light dependent sporangiophore growth shows a near-UV and blue peak, which might originate from flavins with pterins in tight association [144,147–149]. In addition, comparison of flavin and pterin contents of wild-type with those from different photobehavioral mutants show that photoreception of *Phycomyces* requires a balanced and precisely controlled ratio of endogenous flavins and pterins [150,151] These results strongly suggest the involvement of flavin and pterins as near UV and blue light photoreceptor chromophores for phototropism of *Phycomyces*. The blue-light-dependent repair of UV-photodamaged DNA is catalyzed by DNA photolyases, which contain FAD and additionally either a pterin (MTHF; 5,10-methenyl tetrahydrofolate) or 8-hydroxy-5-deazaflavin (8-HDF) as light-harvesting secondary chromophores [146,152].

The involvement of pterin as a photoreceptor pigment in photomovement was initially suggested by the fact that the action spectrum for the phototaxis in *Euglena gracilis* has a major peak near 450 nm, as well as a subsidiary peak near 370 nm [153]. In addition, the action spectrum for the step-up and step-down photophobic response in *Euglena gracilis* showed a high sensitivity in the UV-B/C wavelength region as well as UV-A and blue [154]. Thus, the action spectral UV-A peak itself is ascribable not only to pterin chromophores but also flavin chromophores which have not only blue peaks but also a UV-A and UV-B/C peaks (e.g., PAC and phototropins). Experimental data have also been offered for the involvement of pterins and flavins in photoperception by *Euglena*[155]. Further support for the involvement of pterins in photoperception is provided by the fact that pterin-like fluorescence is associated with proteins of the paraflagellar body [156] and with isolated flagella of *Euglena gracilis*[157,158]. There is also indirect physiological evidence that flavin and pterin-like chromophores are involved in photoperception in the unicellular ciliate *Chlamydodon mnemosyne*, based on the action spectrum of phototaxis. It was shown that phototactic activity was found to be restricted to the blue and near UV range, with a sharp peak at 470 nm and similarly high activity at the near UV end of the spectrum [159]. This result is also supported by evidence that an autofluorescence substance located in the plasma membrane adjacent to stigma is excited by both near ultraviolet (340–380 nm) and blue light (450–480 nm) [160]. Therefore, these results indicate that pterin, as well as flavin, mediate photomovement in response to near UV/blue light. Further interesting evidence has been reported for cyanobacterium *Chlorogloeopsis* PCC 6912. Although its maximum reactivity is confined to the UV-B range, the action spectrum of UV response in *Chlorogloeopsis* suggests that a reduced pterin may serve as the chromophore in the photosensory induction of mycosporine-like amino acid (MAA) [161]. Additional support for this notion came from findings demonstrating that light-dependent induction of mycosporine-like amino acid (MAA) is impaired by the inhibitors of the pterin biosynthetic pathway [161].

### 3.3. The Role of Pterin in the Regulation of Cyanobacterial Phototaxis

In cyanobacteria, the first hint that pterin might play a role in UV-A-induced phototaxis came from analysis of a *pgtA* mutant. To test whether pteridine glucosyltransferase gene (*pgtA*) is involved in UV-A photoperception, phototaxis assays was carried out on soft agar motility plates under lateral UV-A irradiation of various intensities [58]. Interestingly, in the case of wild-type, no oriented movements were observed in response to UV-A at fluence rates up to18 μmol m^−2^s^−1^[58]. Unlike wild-type cells, *pgtA* mutant lost their positive phototaxis but showed negative phototaxis away from the UV-A under conditions of increasing light intensity. These effects were due to the disruption of the *slr1166* gene-encoding pteridine glycosyl transferase, which is required for the final step of tetrahydrocyanopterin biosynthesis [58]. To more precisely determine the role of pterin in UV-A-induced signaling of Syn6803, the effect of pterin biosynthesis inhibitors on phototaxis was investigated. NAS is a specific inhibitor that suppresses sepiapterin reductase [122], the last enzyme of the pathway that forms tetrahydrobiopterin (BH_4_) in cyanobacteria [125,129]. Application of 2 mM NAS inhibited positive phototactic motility of wild-type under UV-A, indicating that final product of sepiapterin reductase, BH_4_-like pterin, participates in positive phototaxis of wild-type cells. In addition, negative phototaxis of the *pgtA* mutant was observed under UV-A in the presence of NAS. This result indicated that the product of the PgtA enzyme, cyanopterin, is involved in the inhibition of the negative phototaxis of wild-type by sensing UV-A. However, inhibition of GTP-cyclohydrolase I (GTPCH), the rate-limiting enzyme for pterin synthesis, with 2,4-diamino-6-hydroxy-pyrimidine (DAHP) potentially increased positive phototaxis and caused a transition of non-motile or negative to positive phototaxis in the wild type and *pgtA* mutant under UV-A, respectively [58]. It provides evidence that in addition to the inhibitory action of cyanopterin containing protein, another unknown pterin compound synthesized by GTPCH might be involved in suppressing positive phototaxis or in facilitating negative phototaxis under UV-A. In this connection, many hitherto studies have reported the involvement of pterins in the photoperception of UV-A and blue light. In the case of the brown algae *Laminaria saccharina*, there is convincing evidence that pterin may be play a role in the photosensing of blue light as a photosynthetic stimulation response [162]. Inhibition of pterin biosynthesis with DAHP resulted in the decrease of pterin detection by HPLC below a control value, as well as a simultaneous reduction of the photosynthetic stimulation response to blue light. Analogously, it can be presumed that MTHF-like unknown pterin participates in photoperception of UV-A/blue light to stimulate negative phototaxis in Syn6803. This hypothesis is reasonably consistent with the recent findings showing that DASH-type cryptochromes contains a highly conserved MTHF-binding site, and hence, utilize this pterin chromophore as a general antenna pigment [163,164]. Several algae, fungi, and *Euglena*, including cyanobacterium, have been proposed to contain different pteridines as possible constituents of UV-A/blue light photoreceptor system [122,161,162]. As a consequence of reduction in pterin content, photoreceptor sensitivity will be reduced to the same extent as the reduced pterin chromophores, as described in previous reports [122,150,162]. Comparable data indicate that inhibitors of pterin biosynthetic enzymes, DAHP and NAS decreased pteridine contents considerably in *Euglena*, *Neurospora* and *Phycomyces*[122]. Therefore, when the sensitivity of UV-A/blue-ight-harvesting pterin chromophore governing negative phototaxis is considerably reduced by DAHP, it seems quite possible that the combined action of both the preexisting intracellular BH_4_ and the PixJ1 (or TaxD1) proteins controlling positive phototaxis may provide Syn6803 cells with properties which invert negative phototaxis to positive.

Classically, comparison of action spectra for relevant responses with absorption or excitation spectra from candidate molecules has provided important insights into the identity of photoreceptors. To further confirm the potential role of pterin for UV sensing in phototaxis, an *in vivo* action spectrum was generated based on the ratio of photon effectiveness of the wild type to that of the *pgtA* mutant. Putative pterin peaks (300, 340 and 380 nm) and an FAD peak (440 nm) were found in the relative action spectrum of positive phototaxis [58,59]. Thus, the prominent peak at around 340–380 nm in the UV-A region is more closely aligned with the UV-A absorbing form of pterins. One possible interpretation of the peak at 340–380 nm is that it represents UV-A absorption by the pterin component of photoreceptor with efficient energy transfer to the flavin chromophore. Therefore, each of the action spectral peaks might well be attributable to the absorption spectral peaks of different pterin chromophores and the flavin chromophore in the UV-A/blue photoreceptor proteins. This fine structure of action spectrum, which might reflect binding properties of the chromophore, presumably pterin, to its apoprotein, provides further evidence for a functional link between the UV-A/blue light sensing and phototaxis in Syn6803. Furthermore, UV-A/blue peaks at 380 and 440 nm obtained from the action spectrum of phototaxis were found to coincide with the fluorescence spectrum of the *in vivo* cyanobacterial cryptochrome DASH (Ccry1), which has both pterin and flavin as the chromophoric group [59]. These results are in agreement with a model in which near UV/blue light-induced responses are mediated by a flavoprotein (cryptochrome-like photoreceptor) containing pterin and flavin [121].

### 3.4. Cyanobacterial Cryptochromes

One of the photoreceptors that mediates responses to UV-A and blue light is cryptochrome [165]. Cryptochromes are flavoprotein photoreceptors that have evolved from photolyases, a class of blue light activated microbial DNA repair enzymes. However, cryptochromes have no photolyase activity. Instead, they use the energy from near-UV/blue light to regulate a variety of growth and adaptive processes in diverse organisms [166]. The photolyase/cryptochrome superfamily includes cyclobutane pyrimidine dimer (CPD) photolyases, the 6-4 pyrimidine pyrimidone (6-4) photolyases, plant and animal cryptochromes, and the so-called “cryptochromes DASH” (newly identified in *Arabidopsis* and *Synechocystis*, homology to CRYS from *Human* and *Drosophila*). All members of this superfamily have an *N*-terminal photolyase-homologous region (PHR) domain. The PHR domain binds a FAD cofactor and a second, light-harvesting chromophore [167,168]. In Syn6803, there exist two open-reading frames (sll1629 and slr0854) with strong sequence similarity and homology to photolyase and cryptochrome. It was shown that *slr0854* encodes a photolyase, whereas *sll1629* has no photolyase activity, indicating that *sll1629* encodes a cryptochrome-type photoreceptor (Cry-DASH) [169,170]. In *Synechocystis*, structural and functional studies suggest that sll1629, a putative cyanobacterial cryptochrome (Cry-DASH) likely functions as a light-responsive transcriptional repressor [171], but the role of this protein as a photoreceptor is not clearly established. Three dimensional structures of the photolyase-like domain from *Arabidopsis thaliana* cryptochrome1 (CRY1) and cryptochrome DASH (Cry-DASH) from Syn6803 have been reported [171,172]. Although the structure retains sufficient space for second chromophores to bind, both structures contain FAD, but the second chromophore seems to be distinct or absent [171]. Recently a representative for an additional class of plant cryptochrome, CRY3, has also been identified as a member of the cry-DASH gene family in *Arabidopsis*[173]. CRY3 is most closely related to Cry-DASH (Sll1629) of Syn6803 [171,173]. CRY3 carries an *N*-terminal sequence, which mediates import into chloroplasts and mitochondria. Biochemical characterization and spectroscopic analysis implies that, like *Escherichia coli* photolyase, both pterin (5,10-methenyl tetrahydrofolate, or MTHF) and FAD bind non-covalently to CRY3 [163,164]. However, the residues responsible for binding of MTHF in CRY3 are not conserved in *Escherichia coli* photolyase, but are strongly conserved in the Cry-DASH subfamily of cryptochromes [163]. In addition, spectroscopic studies on *cry3* mutant that lacks pterin (MTHF) demonstrated pterin (MTHF) to be a functional antenna pigment for the photoreduction of FAD [164]. Similarly, resonance energy transfers from MTHF to FAD were recently demonstrated for the DASH-type cryptochromes, VcCry1 from *Vibrio cholerae*[174]. Therefore, DASH-type cryptochromes are now shown to comprise a highly conserved MTHF binding site, and hence, apparently utilize this MTHF chromophore as a general antenna pigment for efficient energy transfer [163,164,174].

### 3.5. Involvement of Cryptochrome in the Regulation of Phototactic Response to UV-A

The relationship between the role of pterins and cryptochrome in regulating the phototaxis of the Syn6803 was studied using a cryptochrome mutant that had an impaired *ccry1* gene. Although a putative cryptochrome gene, *ccry1* (*sll1629*), was identified in Syn6803 [169], additional information on the *in vivo* function of cyanobacterial cryptochrome Ccry1 has not been reported. Given the close similarities between the cryptochrome sequences of Syn6803 and *Arabidopsis*, Syn6803 cryptochrome Ccry1 is likely to function as a photoreceptor, similar to plant cryptochrome [171,173]. To clarify whether cryptochrome contributes to the phototactic response of Syn6803, we generated a knock-out mutant by disrupting the cryptochrome gene *ccry1*. We compared the phototactic movement of *ccry1* mutant with that of *pgtA* mutant under specific monochromatic lights, including UV-A light. Under UV-A, inactivation of *ccry1* gene in the absence of NAS, like the wild-type, resulted in a non-motile phenotype. Unlike *ccry1* mutant, *pgtA* mutant showed negative phototaxis. However, when tetrahydrobiopterin (BH_4_) synthesis was inhibited by NAS, *ccry1* and *pgtA* mutant exhibited the same phenotype (negative phototaxis), although *pgtA* mutant exhibited notably stronger negative phototaxis than *ccryl* mutant in response to UV-A [59]. These results indicate that Ccry1 and cyanopterin are involved in the regulation of negative phototaxis. It also suggests that cyanopterin could act as a chromophore of cyanobacterial cryptochrome Ccry1. This result is also supported by the fact that the excitation spectrum of *in vivo* cyanobacterial cryptochrome DASH (Ccry1) purified from Syn6803 closely matches the relative action spectrum of phototaxis, based on the ratio of effectiveness of the wild type to that of the *pgtA* mutant at the near UV/blue range [59].

In addition, positive phototaxis of wild-type in UV/blue was completely inhibited by incubation with 2 mM NAS, suggesting that BH_4_-like pterin, formed by sepiapterin reductase (SR), is responsible for induction of positive phototaxis or suppression of negative phototaxis in Syn6803. Whether PixJ1 (or TaxD1)-like protein acts as a real photoreceptor in governing the positive phototaxis of Syn6803 in response to UV-A/blue light, and whether it utilizes BH_4_-containing protein as a regulator for this function remains to be determined. In this respect, it is relevant to note that a possibly positive phototaxis may be mediated via phytochrome-like photoreceptor such as PixJ1 (TaxD1) or BLUF (Slr1694) proteins, as reported in earlier works [54,84–86]. Thus, UV-A appears to affect the negative phototaxis through an unidentified pterin chromophore that is different from phytochrome-dependent chromophores that appear to control positive phototaxis. The above results allow us to postulate that in addition to cyanopterin, one (or two) as yet unidentified UV-absorbing pterin chromophore(s), which may act as an antenna pigment of cryptochrome(s) is responsible for negative phototaxis of Syn6803 in response to near UV-A/blue light. Moreover, there appears to be a UV-A/blue photosensory system that can cause inhibition of negative phototaxis in conjunction with the phytochrome-like proteins such as PixJ1 (TaxD1) and BLUF (Slr1694).

## 4. Possible Role of Pterin as a UV-A-Protecting Compound

A possible role of pterins as UV-protecting compounds has been suggested for the marine planktonic cyanobacterium *Oscillatoria* sp., since UV-A radiation caused a rapid onset of synthesis and massive accumulation of BH_4_ biopterin glucoside, which serves as a protective agent against UV-A illumination [139,140]. Accordingly, in a different context, we cannot exclude the possibility that Syn68003 cells may reduce their susceptibility to UV damage by utilizing pterins as photoprotective compounds similar to the effect of biopterin-glycosides, as observed in marine planktonic cyanobacterium *Oscillatoria* sp. Under UV-A conditions, growth of the UV-A sensitive marine cyanobacterium *Synechococcus* was suppressed, while the UV-A resistant strain *Oscillatoria* grew rapidly owing to the production of high levels of UV-A absorbing biopterin-glycoside [139,140]. From a photoprotection standpoint, the absence of cyanopterin in *pgtA* mutant may have resulted in alternative reasons as to why this mutant is more UV-A sensitive, and shows negative phototaxis. To clarify this question, we measured the difference of spectral photosensitivity in the UV-A region (300–400 nm) between wild-type and *pgtA* mutant at the single cell level, by varying the light intensities (data not shown). Fluence rate-response curves were determined by plotting the phototactic orientation of photoresponsive cells *vs*. light intensities in the UV-A (300–400 nm). Phototactic sensitivity of wild-type cells increased in response to increasing UV-A (300–400 nm) light intensity. In *pgtA* mutant, no obvious changes of phototactic sensitivity were observed, even under high intensities of UV-A, suggesting that the PgtA enzyme product, cyanopterin, is involved in the UV-A light signaling pathway. Therefore, the inability of the *pgtA* mutant to show positive phototactic orientation might be the result of both reduced amounts of pterin and decreased sensitivity of photosensing system in response to UV-A.

## 5. UV Stress and Programmed Cell Death in Cyanobacteria

Cyanobacteria produce reactive oxygen species (ROS) such as superoxide (O_2_^−^), hydroxyl radical (OH^−^) or hydrogen peroxide (H_2_O_2_) which causes oxidative stress during exposure to UV irradiation. UV-induced formation of ROS can severely affect the cell by damaging proteins, cleaving nucleic acids, peroxidizing lipids, and inhibiting photosynthesis; this can ultimately lead to growth inhibition and cell death [11,103]. ROS are known to act as regulators of programmed cell death (PCD) in plant and animal cells, so it is possible that cyanobacteria also undergo apoptosis or programmed cell death (PCD) under UV stress or UV-mediated oxidative stress. UV has been reported to cause apoptotic-like cell death in the unicellular alga *Chlamydomonas reinhardtii* and chlorophyte *Dunaliella viridis*, when subjected to high doses of UV [175,176]. Like chlorophyte *Dunaliella viridis*[176], cyanobacteria may have the capacity to undergo different modes of cell death depending on the various stress factors or their intensity. Experimental evidence for PCD in cyanobacteria was primarily derived from the studies of three species, including the freshwater cyanobacterium *Anabaena* sp., exposed to univalent-cation salts, the bloom-causing cyanobacterium *Microcystis aeruginosa* by treatment with H_2_O_2_ and *Trichodesmium* sp. suffering iron starvation and high light irradiance [177–180]. Recent studies on *Microcystis aeruginosa* showed that H_2_O_2_ induced PCD in a dose-dependent manner and that catalase suppressed the caspase activity, indicating the role of PCD under oxidative stress [178,179]. In oxidative stressed *M. aeruginosa* cells, caspase activity appears to be responsible for the onset of PCD. Metacaspases (MCAs) are cysteine proteinases that have sequence homology to caspases and play important roles in PCD in several prokaryotes, fungi and plants [181–185]. In *Arabidopsis*, metacaspase-8 mediates PCD induced by UV and H_2_O_2_[185]. Caspase-like protease activity was found to be involved in executing PCD in an oceanic nitrogen-fixing cyanobacterium *Trichodesmium* IMS101 [180,186]. This was evidenced by cross-hybridization with human caspase-3 antibodies, increased caspase-like activity with PCD induction, and the inhibition of activity when a caspase-3 specific inhibitor was applied [180]. Analyses of completed genome sequences of cyanobacteria have revealed the widespread presence of metacaspases in some cyanobacteria, including *Anabaena variabilis*, *Gloeobacter violaceus*, *Synechococcus* sp., and *Synechocystis* sp. PCC 6803 [187,188]. Considering the diverse distribution of putative metacaspases genes, the PCD function may have been inherited from the cyanobacteria [189]. Despite increasing literature on PCD events in bacteria and unicellular eukaryotes in response to numerous stresses, little is known about the nature and operational mechanism of UV-induced PCD, or cell death pathways in unicellular photosynthetic organisms which play essential roles in the equilibrium of aquatic ecosystems. The existence of genetically driven cell death phenomena in cyanobacteria and algae indicates that the precise regulation mechanisms involved in the dichotomy between cell survival and cell death were already present in these single-celled organisms. Therefore, further study is necessary to characterize the role of PCD in cyanobacteria and to decipher the important players involved in signaling pathways leading to cell death in response to UV radiation-induced stress. In addition, a more detailed knowledge on PCD in photosynthetic microorganisms may provide new insight into their survival mechanisms, and could delineate the evolutionary origin of cell death processes.

## 6. Concluding Remarks

In recent years, significant progress has been made in identifying the UV-A photoreceptor and several molecular players involved in early events of UV-A signaling pathways in cyanobacteria. However, information about the downstream signaling events and effecter molecules that underlie UV-A induced phototactic responses is very limited. UV-A radiation appears to be involved in a multitude of responses that are summarized as low and high fluence rate-dependent responses. However, further studies will be needed to discriminate between these responses, and to unravel the complexity of UV-A induced signaling systems. Moreover, it remains to be established whether there is functional cross-talk or interaction between these signaling pathways. In general, exposure to high fluence rates of UV-A is likely to cause stress response in cyanobacteria. High fluence rate UV-A-dependent signaling can stimulate the expression of genes involved in negative phototactic responses, and hence, promote the survival of cyanobacteria under UV-A-mediated oxidative stress. Deciphering the complex interplay between signaling components in the regulation of negative phototactic response pathways, and other players of UV-A-induced stress response pathways, provides a valuable insight into the control of cyanobacterial mortality, and an interesting ecological context for defense mechanisms against UV-A. Low fluence rate-dependent UV-A signaling can be mediated by the number of photosensors, including cyanobacterial cryptochrome Cry-DASH (sll1629), cyanobacteriochromes (Cph2 and PixJ1), and signaling molecules such as c-di-GMP and calcium ions. In addition, physiological and genetic analysis of pterin-specific mutant, phototaxis action spectra, and fluorescence spectrum of the *in vivo* cyanobacterial cryptochrome have provided solid support for the hypothesis that pterin can act as a chromophore of putative UV-A/blue photoreceptor. Thus, novel emerging roles of pterin in the UV-A-induced signaling pathway of cyanobacteria merit further investigation. Molecular targets and the regulators of key players and signaling molecules involved in UV-induced signaling pathways have yet to be elucidated. As just one example, characterization of c-di-GMP-binding proteins under UV-A is necessary to broaden our understanding of this signaling mechanism. Future studies will include the identification of new components within these UV-A-induced signal transduction pathways. Biochemical and molecular genetics approaches can be anticipated to identify new “players,” in the same way that the UirS-UirR two-component signaling system was identified as an important component of high fluence rate UV-A-dependent phototactic responses. Furthermore, PCD-like cell death (or that of related proteins) plays an important role in cyanobacteria under conditions of oxidative stress. However, the nature and control mechanisms of PCD under UV stress remain virtually unexplored in cyanobacteria. Thus, advances in our understanding of cellular signaling processes associated with UV-induced cell death and survival strategies in cyanobacteria opens new perspectives on the influence of UV on aquatic ecosystems, and on its impact on population dynamics and photosynthesis.

## Abbreviations

UV: Ultraviolet
ROS: Reactive Oxygen Species
Syn6803: *Synechocystis* sp. PCC 6803
RuBisCO: Ribulose 1,5-Bisphosphate Carboxylase/Oxygenase
CPDs: Cyclobutane Pyrimidine Dimers
CBCRs: Cyanobacteriochromes
PCB: Phycocyanobilin
UirS: UV intensity response Sensor
UirR: UV intensity response Regulator
LsiR: Light and stress integrating response Regulator
HK: Histidine Kinase
ETR: Ethylene Receptor
NAC: *N*-Acetyl Cysteine
c-di-GMP: 3′,5′-cyclic diguanylic acid
cAMP: 3′,5′-cyclic adenosine monophosphate
SYCRP: *Synechocystis* sp. cAMP Receptor Protein
PgtA: Pteridine glycosyltransferase
BGluT: UDP-glucose: BH_4_ α-Glucosyltransferase
BH_4_: Tetrahydrobiopterin
FAD: Flavin Adenine Dinucleotide
GTPCH: GTP Cyclohydrolase I
NAS: *N*-Acetylserotonin
SR: Sepiapterin Reductase
DAHP: 2,4-Diamino-6-hydroxypyrimidine
MAA: Mycosporine-like Amino Acid
BLUF: Sensor of Blue Light using FAD
Cry-DASH: Cryptochrome-*Drosophila*, *Arabidopsis*, *Synechocystis*, *Human*
PHR: Photolyase-Homologous Region
MTHF: 5,10-Methenyl tetrahydrofolate
8-HDF: 8-Hydroxy-5-deazaflavin
PCD: Programmed Cell Death
MCAs: Metacaspases
